# Accelerated Simultaneous T_2_ and T_2_* Mapping of Multiple Sclerosis Lesions Using Compressed Sensing Reconstruction of Radial RARE-EPI MRI

**DOI:** 10.3390/tomography9010024

**Published:** 2023-01-31

**Authors:** Carl J. J. Herrmann, Ludger Starke, Jason M. Millward, Joseph Kuchling, Friedemann Paul, Thoralf Niendorf

**Affiliations:** 1 Berlin Ultrahigh Field Facility (B.U.F.F.), Max Delbrück Center for Molecular Medicine in the Helmholtz Association, 13125 Berlin, Germany; 2 Department of Physics, Humboldt Universität zu Berlin, 12489 Berlin, Germany; 3 Digital Health—Machine Learning Research Group, Digital Health Center, Hasso Plattner Institute, University of Potsdam, 14482 Potsdam, Germany; 4 Experimental and Clinical Research Center, A Joint Cooperation between the Charité Medical Faculty and the Max Delbrück Center for Molecular Medicine in the Helmholtz Association, Campus Berlin-Buch, 13125 Berlin, Germany; 5 NeuroCure Clinical Research Center, Charité—Universitätsmedizin, 10117 Berlin, Germany; 6 Department of Neurology, Charité—Universitätsmedizin, 10117 Berlin, Germany

**Keywords:** MRI, parametric mapping, transversal relaxation time, brain, multiple sclerosis, compressed sensing

## Abstract

(1) Background: Radial RARE-EPI MRI facilitates simultaneous T_2_ and T_2_* mapping (2in1-RARE-EPI). With modest undersampling (R = 2), the speed gain of 2in1-RARE-EPI relative to Multi-Spin-Echo and Multi-Gradient-Recalled-Echo references is limited. Further reduction in scan time is crucial for clinical studies investigating T_2_ and T_2_* as imaging biomarkers. We demonstrate the feasibility of further acceleration, utilizing compressed sensing (CS) reconstruction of highly undersampled 2in1-RARE-EPI. (2) Methods: Two-fold radially-undersampled 2in1-RARE-EPI data from phantoms, healthy volunteers (*n* = 3), and multiple sclerosis patients (*n* = 4) were used as references, and undersampled (R_extra_ = 1–12, effective undersampling R_eff_ = 2–24). For each echo time, images were reconstructed using CS-reconstruction. For T_2_ (RARE module) and T_2_* mapping (EPI module), a linear least-square fit was applied to the images. T_2_ and T_2_* from CS-reconstruction of undersampled data were benchmarked against values from CS-reconstruction of the reference data. (3) Results: We demonstrate accelerated simultaneous T_2_ and T_2_* mapping using undersampled 2in1-RARE-EPI with CS-reconstruction is feasible. For R_extra_ = 6 (TA = 01:39 min), the overall MAPE was ≤8% (T_2_*) and ≤4% (T_2_); for R_extra_ = 12 (TA = 01:06 min), the overall MAPE was <13% (T_2_*) and <5% (T_2_). (4) Conclusion: Substantial reductions in scan time are achievable for simultaneous T_2_ and T_2_* mapping of the brain using highly undersampled 2in1-RARE-EPI with CS-reconstruction.

## 1. Introduction

Brain lesions in multiple sclerosis (MS) have MRI characteristics that can be used to aid diagnosis and to discriminate among different lesion types. Given these features, parametric mapping of multiple MR contrasts would be highly clinically relevant [1,2,3]. Nevertheless, routine clinical practice demands that scan acquisition times are kept to a minimum. It is therefore conceptually appealing to establish methods to simultaneously acquire multiple MR contrasts, and to explore approaches to accelerate this as much as possible. To realize this, we previously presented a radially-sampled RARE-EPI hybrid that facilitates simultaneous T_2_ and T_2_* mapping (2in1-RARE-EPI) [4]. Similar to previously proposed RARE-EPI combined-acquisition-techniques [5,6,7], the MR signal in 2in1-RARE-EPI is acquired with a RARE module followed by an EPI module to capture the T_2_ and T_2_* decay simultaneously after each excitation. The radial sampling of k-space data implemented in 2in1-RARE-EPI promotes acceleration through undersampling. This approach reduces scan time and eliminates the risk of slice misregistration, as the T_2_ and T_2_* maps are inherently co-registered. In general, the benefits of quantitative mapping come with the drawback of increased scan time, since multiple images with different contrast weightings need to be acquired. This limits the implementation of quantitative mapping in routine clinical practice and broader clinical studies. In our previous study, the acquisition time for simultaneous T_2_ and T_2_* mapping was reduced to 77% of the comparable reference methods Multi-Spin-Echo (MSE) and Multi-Gradient-Recalled-Echo (MGRE) due to the use of the hybrid acquisition and a modest radial undersampling factor (R = 2) [4]. Unlike previous methods for simultaneous T_2_ and T_2_* mapping, such as Spin- and Gradient-Echo (SAGE) [8,9,10,11] and 3D Echo Planar Time-Resolved Imaging (3D EPTI) [12], which utilize multiple EPI readouts before and after 180° refocusing pulses to obtain multiple images with different contrast weightings for T_2_ and T_2_* mapping, 2in1-RARE-EPI samples k-space spokes in a hybrid acquisition in an echo train consisting of a RARE module followed by an EPI module.

Additional reduction in scan time will further enhance patient comfort and compliance, and is a fundamental precursor for broader clinical studies on the potential of T_2_ and T_2_* as imaging biomarkers in MS, including application in a drug trial [13]. A promising approach to accelerate quantitative mapping in MRI is the use of compressed sensing (CS) reconstruction of highly undersampled data [14,15,16,17,18]. CS has revolutionized MRI by exploiting the inherent sparsity of natural images to allow high k-space undersampling factors [19,20]. First commercial applications have recently achieved clinical approval [21,22,23]. Previous contributions have shown accelerated quantitative measurements in vivo for T_2_ mapping in cartilage and liver [18], single point imaging T_2_* mapping of a mouse tumor [15], and T_2_ mapping of the mouse heart [14]. However, little has been reported to confirm the effectiveness of CS for relaxation time mapping and to evaluate the effect of undersampling rates on the reliability of quantitative mapping of neurodegenerative and neuroinflammatory disease in vivo.

Here, we demonstrate the first application of CS reconstruction for simultaneous T_2_ and T_2_* mapping with accelerated 2in1-RARE-EPI, in phantoms, healthy volunteers, and MS patients, and provide practical recommendations.

## 2. Materials and Methods

### 2.1. MR Data Acquisition

**Figure 1:**
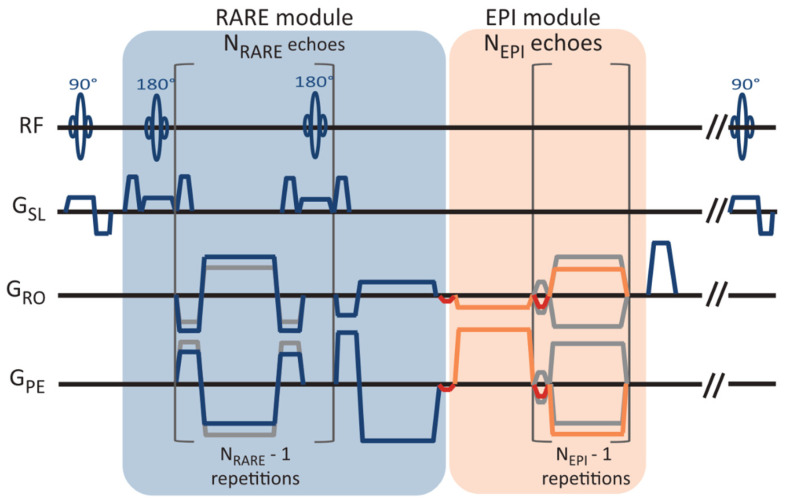
Pulse sequence diagram of the radially-sampled RARE-EPI hybrid, which facilitates simultaneous T_2_ and T_2_* mapping (2in1-RARE-EPI). The magnetic resonance signal is first acquired with a RARE module with N_RARE_ echoes, followed by the acquisition with an EPI module of N_EPI_ echoes. N_RARE_ = 14 and N_EPI_ = 18 were used in this study. The transition between the spokes in the EPI module are realized with small blip gradients (marked in red).

In 2in1-RARE-EPI, the MR signal is first acquired with a RARE module (T_2_ decay), followed by the acquisition with an EPI module (T_2_* decay). A pulse sequence diagram of 2in1-RARE-EPI is depicted in Figure 1. In the EPI module, transitions between the k-space spokes are realized by small blip gradients.

The MR data were acquired with 2in1-RARE-EPI at 3.0 T (Siemens Magnetom SkyraFit, Erlangen, Germany, maximum gradient strength, 43 mT/m; maximum slew rate 180.18 mT/m/ms) using the body RF coil for transmission and a 32-channel head RF coil (Siemens, Erlangen, Germany) for signal reception. The radial k-space data were corrected for gradient delays based on a calibration scan acquired prior to each scan (TA_Calib_ = 32 s). The following imaging parameters were used: FOV = (232 × 232) mm^2^, matrix size = 256 × 256, slice thickness = 5 mm, no. of slices = 3, TR = 2000 ms, N_RARE_ = 14, N_EPI_ = 18, echo-spacing (RARE/EPI) = 6.5 ms/2.3 ms, receiver bandwidth = 610 Hz/px, no. of shots (N_S_) = 200, acquisition time (TA) = TA_Calib_ + N_S_*TR = 07:12 min. For the phantom study, a single slice and FOV = (212 × 212) mm^2^ were used. The acquisition of 200 shots with a matrix size of 256 × 256 corresponds to an undersampling factor of R = 2 for the TE images.

### 2.2. Phantom Study

The phantom was designed to mimic the T_2_ and T_2_* of brain tissue, and contained 12 plastic tubes (volume = 15 mL, diameter = 15 mm), filled with water-based solutions of the iron oxide nanoparticle-based contrast agent Resovist (Schering, Berlin, Germany). The iron oxide nanoparticles decrease T_2_ and T_2_* of the solvent, depending on their concentration in the solution. For the phantom, different solutions with iron concentrations ranging between 2.3 to 12.3 μg Fe/mL were used. Specifically, the tubes were filled with solutions of the following iron concentrations (from left to right and top to bottom): 5.9, 4.6, 6.15, 6.15, 4.01, 4.01, 2.95, 2.95, 2.3, 2.3, 12.3, 8.02 μg Fe/mL and 3.51 μg Fe/mL outside the tubes.

### 2.3. In Vivo Study

The in vivo feasibility study included three healthy volunteers (1 female/2 male, age = 31–46 years, body mass index (BMI) = 23.6–25.9 kg/m^2^) and four MS patients (3 female/1 male, age = 30–39 years, BMI = 22.3–34.9 kg/m^2^). For the in vivo study including healthy volunteers, three slices were acquired, which covered the lateral ventricles. For the in vivo study including MS patients, three slices covering MS specific lesions were acquired, which were selected based on a 2D T_2_-weighted RARE scan.

### 2.4. T_2_ and T_2_* Mapping

In 2in1-RARE-EPI, the MR signal is first acquired with a RARE module (T_2_ decay), followed by the acquisition with an EPI module (T_2_* decay). For T_2_ and T_2_* mapping, a linear least-square fit was applied to the images reconstructed from k-space data acquired at the same echo times (TEs) within the echo train. For T_2_ mapping, the first echo was excluded from the fit to limit the effect of stimulated echoes. This approach is generally effective at reducing stimulated echo contamination, however, it may still result in erroneous estimations of T_2_, particularly for very low T_2_ values [24].

### 2.5. Undersampling, Data Pre-Processing, and CS-Reconstruction

The two-fold radially-undersampled 2in1-RARE-EPI data, used as reference, were undersampled at each TE by removing every nth k-space spoke. Undersampling factors of R_extra_ = 1, 2, 4, 6, 8, 10, and 12 were investigated. This undersampling scheme corresponds to an effective acceleration of R_eff_ = 2–24. For each receive channel, non-regularized reconstructions of the first TE images were obtained with the Michigan Image Reconstruction Toolbox (MIRT) NUFFT [25,26] for the RARE and EPI modules. Based on these images, coil sensitivities were computed with the ESPIRiT method [27,28] implemented in the Berkeley Advanced Reconstruction Toolbox (BART, v0.7.00) [29,30,31] for each undersampling factor and module. K-space noise levels and correlations were estimated from background regions in the 1D inverse Fourier transform along the frequency encoding direction. Noise pre-whitening using the Cholesky decomposition of the inverse covariance matrix was performed [32,33]. Individual echo parallel imaging CS-reconstructions were computed with wavelet regularization using the Fast Iterative Soft-Thresholding Algorithm implemented in BART [34,35,36,37]. Automatic optimization of the regularization strength (λ) is essential to ensure reproducibility and reliability of the results [38]. We employed an adapted version of the discrepancy principle [39,40,41]. Given radial non-uniform Fourier transform $F_{\mathrm{nuft}}$ and coil sensitivities *S*, the expected quadratic deviation of a true, but unknown, image *r*_t_ from the measured data *y* is

(1) $$E\left[\|y-F_{\mathrm{nuft}}Sr_{t}\|{}_{2}^{2}\right]=2n_{\mathrm{fe}}n_{s}n_{c}\sigma^{2}=\epsilon ,$$

where $n_{\mathrm{fe}}$ is the number of frequency encoding points, $n_{s}$ the number of spokes, $n_{c}$ the number of channels, and σ the noise standard deviation in the real and imaginary channels. The discrepancy principle consists of finding a value of *λ* such that

(2) $$\|y-F_{\mathrm{nuft}}Sr\|{}_{2}^{2}\approx \epsilon \;$$

for CS-reconstruction r(λ). However, implementation of the discrepancy principle, and the CS-reconstruction in general, requires use of a non-uniform fast Fourier transform ($F_{\mathrm{nufft}}$) to limit computation times, and data-derived coil sensitivities $\tilde{S}$, both of which introduce errors, such that the minimal attainable deviation exceeds the noise level for high SNR data:

(3) $$\underset{r}{\min}\|y-F_{\mathrm{nufft}}\tilde{S}r\|{}_{2}^{2}>\epsilon .\;$$

The NUFFT and coil sensitivity induced errors are proportional to the signal level, and thus highest in the k-space center, while the measurement noise is uniformly distributed. We exploited this fact by considering only the k-space periphery in the computation of the deviation of the reconstruction from the measured data. For this purpose, we selected only the highest $n_{r}=64$ spatial frequencies on each spoke using masking matrix R. The discrepancy principle condition thus becomes

(4) $$\|Ry-RF_{\mathrm{nufft}}\tilde{S}r\|{}_{2}^{2}\approx 2n_{r}n_{s}n_{c}\sigma^{2}\cdot \eta =\epsilon^{\prime },$$

where η is an additional factor to fine tune the degree of smoothing, which was set to 0.97 in our study [41]. Importantly, this masking is only applied during the computation of the deviation from the data and not during the CS-reconstruction itself. The Illinois algorithm was employed to find the fitting value of *λ* with a tolerance of 0.1% in the data deviation. Using this adapted form of the discrepancy principle, high reconstruction quality could be achieved with automatic tuning for most images. The above condition could not be met for the highest SNR echoes in the RARE module, and for the first echoes of the EPI module without retrospective undersampling. In these cases, a small, fixed *λ* value of 2.5 × 10^−5^ was employed to ensure the reduction of aliasing artifacts with only minimal deviation from the measured data.

### 2.6. Assessment of the Effect of Acceleration on T_2_ and T_2_* Mapping

**Figure 2:**
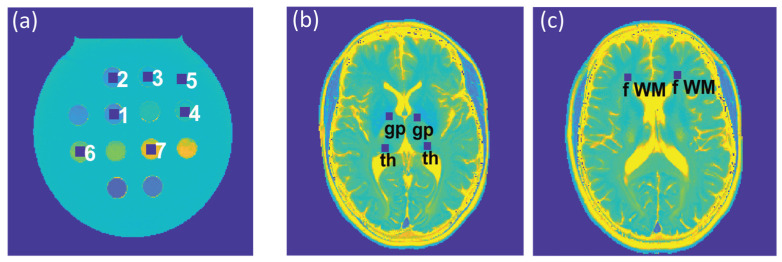
(**a**) Regions of interest (ROIs) (size: 9 × 9 pixels), corresponding to tubes with varying iron concentrations in the phantom, which were used for the regression and Bland–Altman plot analyses and the calculation of the median absolute percentage error (MAPE). (**b**,**c**) ROIs (size: 7 × 7 pixels) used for further analysis of the in vivo study involving healthy volunteers were placed within the globus pallidus (gp) and thalamus (th) (**b**) and periventricular frontal white matter (fWM) (**c**), in the left and right hemispheres.

To assess the feasibility of undersampling, regression and Bland–Altman plot analyses were performed for every undersampling factor. T_2_ and T_2_* derived from CS-reconstruction of the undersampled data were benchmarked against T_2_ and T_2_* obtained from CS-reconstruction of the reference data (R_extra_ = 1). For analysis of the phantom study, seven ROIs (size = 9 × 9 pixels) were placed within the phantom (Figure 2a), corresponding to the position of the plastic tubes containing the varying iron concentrations. For analysis of the T_2_ and T_2_* maps obtained from healthy volunteers, six ROIs (size = 7 × 7 pixels) were selected for each subject, which were placed within the following anatomical brain regions: the globus pallidus, thalamus, and frontal (periventricular) white matter, in both, the left and right hemisphere (Figure 2b,c). For analysis of the T_2_ and T_2_* maps obtained from the patient cohort, eight ROIs (size = 7 × 7 pixels) were selected to cover MS lesions identified in four patients.

Multiple linear regression was used to assess the agreement between the reference and undersampled data, which also takes into account the effects of ROI and subject, and their interaction. For the Bland–Altman plot analyses, the median of the T_2_ and T_2_* differences, $M\left(\Delta T_{2}\right)$ and $M\left(\Delta T_{2}^{*}\right)$ and the interquartile range (IQR) were used to calculate the limits-of-agreement (LOAs) according to $LOAs=M\left(\Delta T_{2}^{\left(*\right)}\right)\pm 1.45\cdot IQR$. since it was observed that the differences of T_2_ and T_2_* values among the reference and undersampled data did not follow a Gaussian distribution in all cases.

Furthermore, the median absolute percentage error (MAPE) of T_2_ and T_2_* derived from undersampled 2in1-RARE-EPI was calculated relative to the corresponding T_2_ and T_2_* deduced from the CS-reconstruction of the 2in1-RARE-EPI reference (R_extra_ = 1) according to:

(5) $$\mathrm{MAPE}\;\mathrm{of}{\;T}_{2}^{\left(*\right)}\left(R_{\mathrm{extra}}\right)=M\left(\frac{T_{2}^{\left(*\right)}\left(R_{\mathrm{extra}}\right)-T_{2}^{\left(*\right)}}{T_{2}^{\left(*\right)}}\cdot 100\right),$$

where M denotes the median and T_2_^(^*^)^(R_extra_) and T_2_^(^*^)^ the T_2_ and T_2_* values derived from 2in1-RARE-EPI with and without retrospective undersampling, respectively. The calculation of the MAPE was performed for the same ROIs used for the regression and Bland–Altman plot analyses. Differences in MAPE among R_extra_ factors were analyzed using the non-parametric repeated-measures Friedman test, followed by the Dunn’s post-hoc test with the Benjamini–Hochberg correction for multiple comparisons. Data were analyzed using R v.3.6.3.

## 3. Results

**Figure 3:**
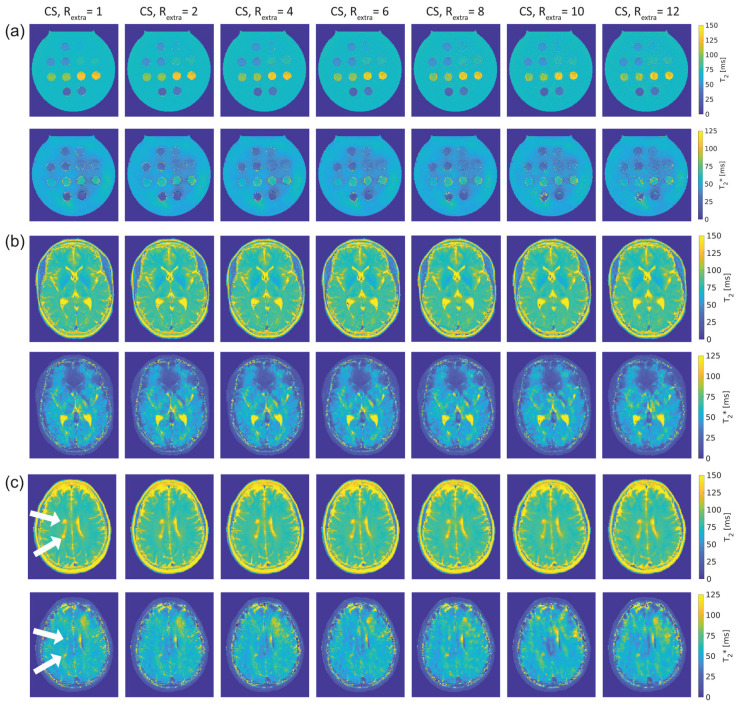
T_2_ and T_2_* maps obtained from 2in1-RARE-EPI using extra undersampling factors of R_extra_ = 1–12 (R_extra_ = 1 is used as a reference). The undersampling factor R_extra_ increases from left to right. (**a**): T_2_ (top) and T_2_* (bottom) maps of a phantom containing tubes filled with solutions of different iron concentration, which mimics typical T_2_ and T_2_* values of brain tissue. (**b**): T_2_ (top) and T_2_* (bottom) maps covering the lateral ventricles of the brain of a healthy 30-year-old female volunteer. (**c**): T_2_ (top) and T_2_* (bottom) maps depicting two periventricular lesions in the right cerebral hemisphere of a 30-year old female multiple sclerosis (MS) patient (white arrows).

Figure 3 shows T_2_ and T_2_* maps of the phantom and in vivo studies obtained with 2in1-RARE-EPI for R_extra_ = 1–12. Figure 3a shows the T_2_ and T_2_* maps of the phantom. For the T_2_ maps, increasing the undersampling rate to R_extr_ ≥ 6 (TA reduced to ≤01:39 min) resulted in a minor increase in T_2_ for low iron concentrations (T_2_(R_extra_) ≥ 100 ms). Despite this, no visible change in the T_2_ maps was apparent. In the T_2_* maps, undersampling artifacts were apparent at R_extra_ = 8 and became more pronounced with increasing undersampling.

Figure 3b shows T_2_ and T_2_* maps of the brain of a healthy 30-year-old female volunteer. Increasing undersampling to R_extra_ = 10 (TA reduced to 01:12 min) resulted in a minor reduction of high spatial frequency information, which became apparent in the small gyri of the brain.

The T_2_ and T_2_* maps of the brain of a 30-year-old female MS patient are depicted in Figure 3c. As in the case of the healthy volunteers, increasing undersampling to R_extra_ = 10 resulted in a minor reduction of high spatial frequency information. The periventricular MS lesions were clearly delineated up to R_extra_ = 12 in the T_2_ maps. In T_2_* maps, these lesions were clearly delineated up to R_extra_ = 10.

**Figure 4:**
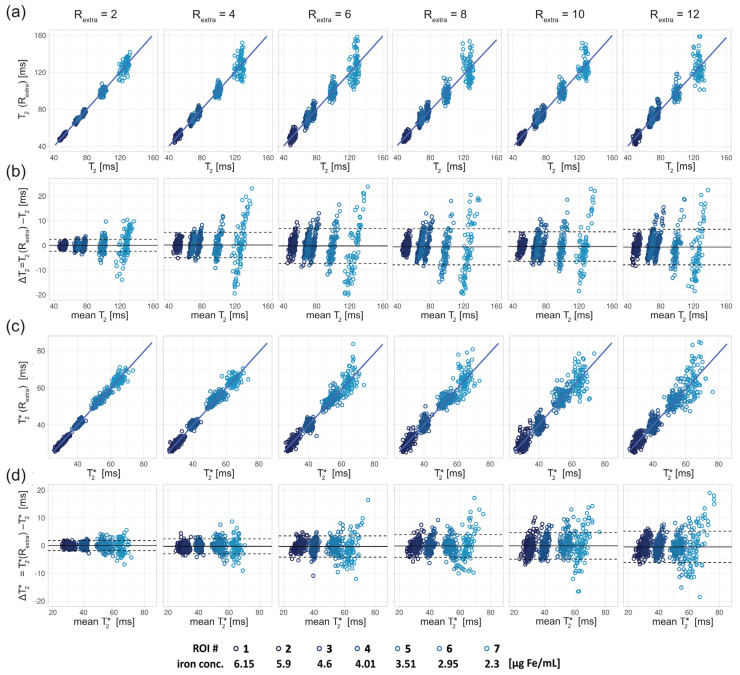
Scatter and Bland–Altman plots of T_2_ and T_2_* calculated from seven ROIs (size: 9 × 9 pixel) placed within the phantom (corresponding to seven different iron concentrations) for undersampling factors R_extra_ = 2–12, increasing from left to right. (**a**) Scatter plots comparing T_2_ derived from retrospectively undersampled 2in1-RARE-EPI, denoted T_2_ (R_extra_), against T_2_ obtained from the 2in1-RARE-EPI reference (R_extra_ = 1), denoted T_2_. (**b**) Bland–Altman plots comparing T_2_ derived from retrospectively undersampled 2in1-RARE-EPI, denoted T_2_ (R_extra_), against T_2_ obtained from the 2in1-RARE-EPI reference (R_extra_ = 1), denoted T_2_. (**c**) Scatter plots comparing T_2_* derived from retrospectively undersampled 2in1-RARE-EPI, denoted T_2_* (R_extra_), against T_2_* obtained from the 2in1-RARE-EPI reference (R_extra_ = 1), denoted T_2_*. (**d**) Bland–Altman plots comparing T_2_ derived from retrospectively undersampled 2in1-RARE-EPI, denoted T_2_* (R_extra_), against T_2_* obtained from the 2in1-RARE-EPI reference (R_extra_ = 1), denoted T_2_*. In the scatter plots, the regression line obtained from multiple linear regression is depicted as a solid line. In the BA plots, the median of the T_2_/T_2_* differences and the limits-of-agreement are depicted as solid and dashed lines, respectively.

**Figure 5:**
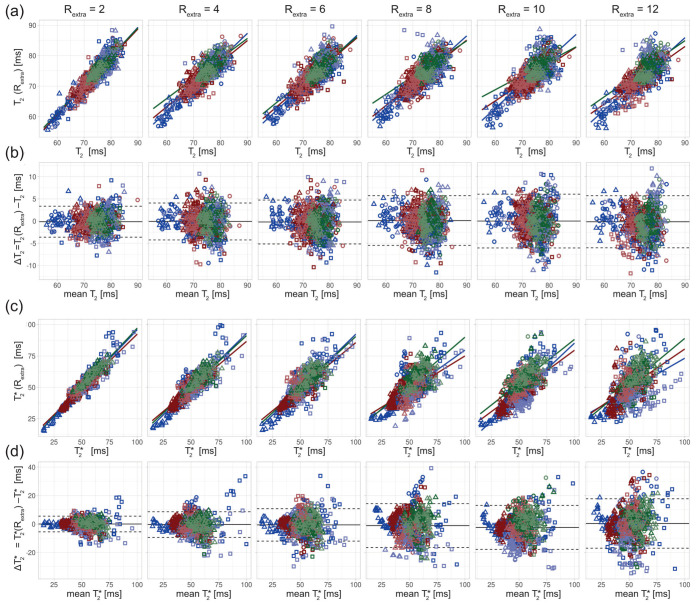
Scatter and Bland–Altman plots of T_2_ and T_2_* calculated from brain scans of 3 healthy volunteers (denoted by square, circle, and triangle) for extra undersampling factors R_extra_ = 2–12, increasing from left to right. The color of the data points denotes the anatomical locations of the ROIs (size: 7 × 7 pixel, blue: thalamus; red: globus pallidus; green: frontal white matter; dark/light = left/right hemisphere). (**a**) Scatter plots comparing T_2_ derived from retrospectively undersampled 2in1-RARE-EPI, denoted T_2_ (R_extra_), against T_2_ obtained from the 2in1-RARE-EPI reference (R_extra_ = 1), denoted T_2_. (**b**) Bland–Altman plots comparing T_2_ derived from retrospectively undersampled 2in1-RARE-EPI, denoted T_2_ (R_extra_), against T_2_ obtained from the 2in1-RARE-EPI reference (R_extra_ = 1), denoted T_2_. (**c**) Scatter plots comparing T_2_* derived from retrospectively undersampled 2in1-RARE-EPI, denoted T_2_* (R_extra_), against T_2_* obtained from the 2in1-RARE-EPI reference (R_extra_ = 1), denoted T_2_*. (**d**) Bland–Altman plots comparing T_2_ derived from retrospectively undersampled 2in1-RARE-EPI, denoted T_2_* (R_extra_), against T_2_* obtained from 2in1-RARE-EPI reference (R_extra_ = 1), denoted T_2_*. In the scatter plots, separate regression lines for each anatomical region are depicted (solid lines), which were obtained from multiple linear regression. In the BA plots, the median of the T_2_/T_2_* differences and the limits-of-agreement are depicted as solid and dashed lines, respectively.

**Figure 6:**
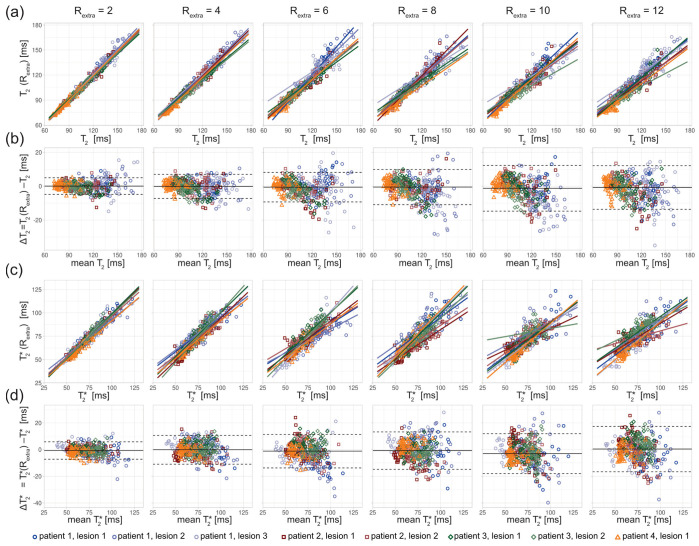
Scatter and Bland–Altman plots of T_2_ and T_2_* calculated from eight ROIs covering lesions in three MS patients, for undersampling factors R_extra_ = 2–12, increasing from left to right. (**a**) Scatter plots comparing T_2_ derived from retrospectively undersampled 2in1-RARE-EPI, denoted T_2_ (R_extra_), against those obtained from the 2in1-RARE-EPI reference (R_extra_ = 1), denoted T_2_. (**b**) Bland–Altman plots comparing T_2_ derived from retrospectively undersampled 2in1-RARE-EPI, denoted T_2_ (R_extra_), against T_2_ obtained from the 2in1-RARE-EPI reference (R_extra_ = 1), denoted T_2_. (**c**) Scatter plots comparing T_2_* derived from retrospectively undersampled 2in1-RARE-EPI, denoted T_2_* (R_extra_), against T_2_*obtained from the 2in1-RARE-EPI reference (R_extra_ = 1), denoted T_2_*. (**d**) Bland–Altman plots comparing T_2_* derived from retrospectively undersampled 2in1-RARE-EPI, denoted T_2_* (R_extra_), against T_2_* obtained from the 2in1-RARE-EPI reference (R_extra_ = 1), denoted T_2_*. In the scatter plots, separate regression lines for each lesion are depicted (solid lines), which were obtained from multiple linear regression. In the BA plots, the median of the T_2_/T_2_* differences and the limits-of-agreement are depicted as solid and dashed lines, respectively.

For closer examination, the results of the regression and Bland–Altman analyses are depicted in Figure 4, Figure 5 and Figure 6.

For the phantom study, T_2_ depicted in the scatter (Figure 4a) and Bland–Altman plots (Figure 4b) showed increased variance with increasing undersampling. The multiple linear regression showed a decrease in the adjusted R^2^ with increased undersampling for T_2_ (0.99, 0.97, 0.92, 0.94, 0.93, 0.92 for R_extra_ = 2–12, respectively) and T_2_* (0.99, 0.99, 0.96, 0.95, 0.94, 0.89 for R_extra_ = 2–12, respectively). This corresponds to a decrease of the adjusted R^2^ of 7% and 10% for the T_2_ and T_2_*, respectively, when increasing R_extra_ from 2 to 12. For T_2_, a minor increase of the limits-of-agreement (LOAs) from −2.35 ms and 2.46 ms at R_extra_ = 2 to −7.41 ms and 6.16 ms at R_extra_ = 12 (TA reduced to 01:06 min) was observed. The median of the T_2_ differences showed a minor decrease from 0.05 ms at R_extra_ = 2 to −0.62 ms at R_extra_ = 12. These results show that 2in1-RARE-EPI-based T_2_ mapping supports high undersampling in conjunction with CS reconstruction. For T_2_*, the scatter plots (Figure 4c) and Bland–Altman plots (Figure 4d) revealed an increase in the variance. T_2_* showed a minor increase of the LOAs from −1.80 ms and 1.81 ms at R_extra_ = 2 to −6.07 ms and 5.07 ms at R_extra_ = 12. The median of the T_2_* differences decreased from −0.01 ms at R_extra_ = 2 to −0.50 ms at R_extra_ = 12. These results demonstrate that 2in1-RARE-EPI-based T_2_* mapping supports high undersampling in conjunction with CS reconstruction.

The in vivo study in healthy volunteers showed increased variance of T_2_ and T_2_* with increasing undersampling (Figure 5). The multiple linear regression showed a decrease in the adjusted R^2^ with increased undersampling for T_2_ (0.85, 0.79, 0.72, 0.69, 0.67, 0.70 for R_extra_ = 2–12, respectively) and T_2_* (0.90, 0.79, 0.71, 0.59, 0.71, 0.41 for R_extra_ = 2–12, respectively). This corresponds to a decrease of the adjusted R^2^ of 18% and 54% for the T_2_ and T_2_*, respectively, when increasing R_extra_ from 2 to 12. The adjusted R^2^ of T_2_* at R_extra_ = 8 is decreased by 34% relative to R_extra_ = 2. The results of the Bland–Altman plot analysis of T_2_ showed a minor increase of the LOAs from −3.62 ms and 3.36 ms at R_extra_ = 2 to −6.00 ms and 5.71 ms at R_extra_ = 12. The median of the T_2_ differences was −0.13 ms at R_extra_ = 2 and −0.15 at R_extra_ = 12. The Bland–Altman plot analysis of T_2_* showed an increase of the LOAs from −5.48 ms and 5.55 ms at R_extra_ = 2 to −16.95 ms and 17.76 ms at R_extra_ = 12. The median of the T_2_* differences increased from −0.04 ms at R_extra_ = 2 to 0.40 ms at R_extra_ = 12.

The in vivo study in MS patients revealed an increase in the T_2_ variance with increasing undersampling for the ROIs covering the lesions (Figure 6). The scatter plots show a decrease of the slope of the regression lines with increased undersampling for T_2_ and T_2_* (Figure 6a,c). The multiple linear regression showed a decrease in the adjusted R^2^ with increased undersampling for T_2_ (0.98, 0.97, 0.94, 0.92, 0.91, 0.92 for R_extra_ = 2–12, respectively) and T_2_* (0.94, 0.88, 0.80, 0.81, 0.73, 0.75 for R_extra_ = 2–12, respectively). This corresponds to a decrease of the adjusted R^2^ of 6% and 20% for the T_2_ and T_2_*, respectively, when increasing R_extra_ from 2 to 12. The Bland–Altman plot analysis showed an increase of the LOAs from −4.86 ms and 4.94 ms at R_extra_ = 2 to −13.65 ms and 12.09 ms at R_extra_ = 12. The median decreased from −0.04 ms at R_extra_ = 2 to −0.78 ms at R_extra_ = 12. For T_2_*, the LOAs obtained from the Bland–Altman analysis increased from −7.27 ms and 5.76 ms at R_extra_ = 2 to −16.67 ms and 17.42 ms at R_extra_ = 12. The median of the T_2_* differences increased from −0.75 ms at R_extra_ = 2 to 0.37 ms at R_extra_ = 12.

**Figure 7:**
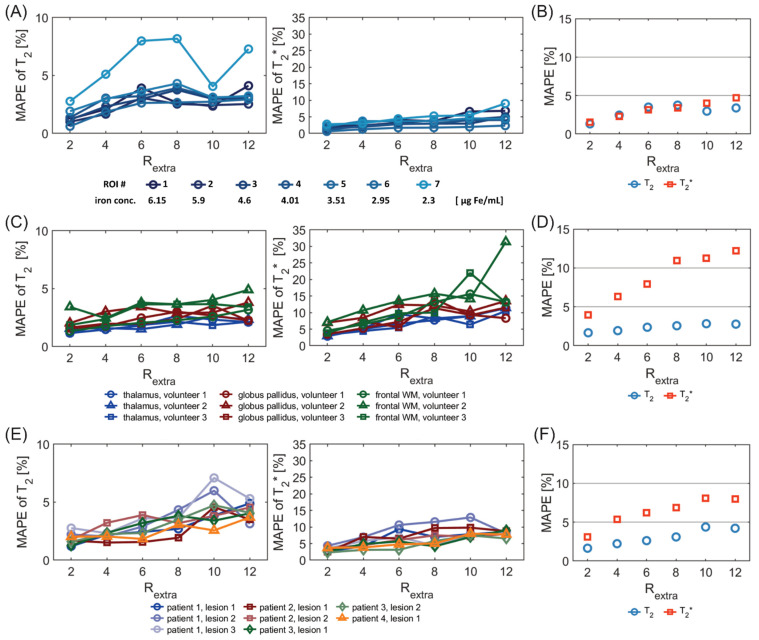
MAPE of T_2_ and T_2_* derived from undersampled 2in1-RARE-EPI (R_extra_ = 2–12), relative to the corresponding T_2_ and T_2_* values obtained from the 2in1-RARE-EPI reference (R_extra_ = 1). MAPEs were calculated for the same ROIs as used for the scatter and Bland–Altman plot analysis (Figure 2, Figure 3 and Figure 4). (**A**) MAPE of T_2_ (left) and T_2_* (right) for seven ROIs placed within the phantom (corresponding to seven different iron concentrations) (**B**) Overall MAPE of T_2_ and T_2_* comprising all seven ROIs placed within the phantom. (**C**) MAPE of T_2_ and T_2_* values derived from brain scans of 3 healthy volunteers. The MAPE was calculated based on the combined ROIs of the left and right hemisphere for each brain region. (**D**) Overall MAPE of T_2_ and T_2_* comprising all ROIs and all healthy volunteers. (**E**) MAPE of T_2_ and T_2_* for eight ROIs covering lesions in four MS patients. (**F**) Overall MAPE of T_2_ and T_2_* comprising all ROIs covering the eight lesions.

Figure 7A shows the MAPE analysis of T_2_ and T_2_* for the phantom study. There was a significant difference in the MAPE of T_2_ of the 7 ROIs placed within the phantom among different undersampling factors R_extra_ (*p* = 3.8 × 10^−5^, Friedman test). There were significant differences of the MAPE of T_2_ between R_extra_ = 6, 8, and 12 and R_extra_ = 2 (*p* = 4.7 × 10^−3^, 3.2 × 10^−3^, 2.1 × 10^−3^, respectively, Dunn’s post-hoc test). The overall MAPE of T_2_ (Figure 7B) for R_extra_ = 8 was only 3.7%. For T_2_*, there were significant differences in the MAPE among the various undersampling factors (*p* = 3.1 × 10^−6^). There were significant differences between R_extra_ = 10–12 and R_extra_ = 2 (2.3 × 10^−2^, 4.6 × 10^−4^, respectively). The overall MAPE of T_2_* (Figure 7B) for R_extra_ = 12 was 4.7%.

Figure 7C shows the MAPE of T_2_ and T_2_* for the in vivo study with healthy volunteers. For the MAPE of T_2_, significant differences were obtained among different undersampling factors R_extra_ (*p* = 1.0 × 10^−4^) for the six ROIs placed in the brain. There were significant differences of the MAPE of T_2_ between R_extra_ = 8–12 and R_extra_ = 2 (*p* = 7.5 × 10^−3^, 5.3 × 10^−3^, 7.2 × 10^−3^, respectively). This shows the consistency of the T_2_ measurement within subjects for R_extra_ below 8. For T_2_*, there were significant differences in the MAPE among the various undersampling factors (*p* = 7.3 × 10^−7^). There were significant differences of the MAPE of T_2_* between R_extra_ = 6–12 and R_extra_ = 2 (*p* = 1.0 × 10^−2^, 2.4 × 10^−4^, 3.1 × 10^−4^, 3.2 × 10^−5^). This shows the consistency of the T_2_* measurement within subjects for R_extra_ below 6. The overall MAPE of T_2_ and T_2_*(Figure 7D) for R_extra_ = 6 was as small as 2.4% and 7.9%, respectively.

Figure 7E shows the MAPE of T_2_ and T_2_* for the in vivo study with MS patients. There was a significant difference of the MAPE of T_2_ values of the eight ROIs, covering MS lesions, among different undersampling factors R_extra_ (*p* = 1.5 × 10^−5^). There were significant differences of the MAPE of T_2_ between R_extra_ = 8–12 and R_extra_ = 2 (*p*= 1.0 × 10^−2^, 1.7 × 10^−4^, 1.0 × 10^−4^, respectively). This shows the consistency of the T_2_ measurement within subjects for R_extra_ below 8. For T_2_*, there were significant differences in the MAPE among the various undersampling factors (*p* = 1.8 × 10^−4^). There were significant differences of the MAPE of T_2_* between R_extra_ = 6–12 and R_extra_ =2 (*p* = 9.4 × 10^−3^, 6.7 × 10^−3^, 7.4 × 10^−5^, 7.7 × 10^−5^, respectively_,_). This shows the consistency of the T_2_* measurement within subjects for R_extra_ below 6. The overall MAPE of T_2_ and T_2_* (Figure 7F) for R_extra_ = 6 was 2.6% and 6.2%, respectively.

## 4. Discussion

In this study, we demonstrated the feasibility of highly accelerated simultaneous T_2_ and T_2_* mapping using a radially-undersampled RARE-EPI hybrid in conjunction with CS-reconstruction. The findings from this feasibility study support the application of quantitative T_2_ and T_2_* mapping of the brain in clinical practice, which could be utilized for broader clinical studies on the potential of T_2_ and T_2_* as imaging biomarkers in MS.

The regression and Bland–Altman analysis revealed an increase of the variance of the T_2_ and T_2_* values with increasing undersampling for both the phantom experiments and the in vivo studies. The Bland–Altman analysis showed that the median of the differences of T_2_ and T_2_* values were close to zero in all cases, for all investigated undersampling factors, which suggests that an increase in undersampling does not introduce a systematic bias in T_2_ and T_2_*. 

The higher variance in ROIs 6 and 7 can be attributed to the fact that there is a large and sharp signal drop at the edge of the plastic tubes for these ROIs due to the difference in T_2_ values of the solution within the tube and that in the surrounding body of the phantom. This leads to increased variance of T_2_ values caused by more pronounced Gibbs ringing artifacts. Furthermore, residual aliasing artifacts which are not fully compensated by the CS reconstruction might have a higher impact on ROIs 6 and 7, as the mislocated signal stemming from the phantom body deviates more in T_2_/T_2_* than for the other ROIs.

In addition, the higher variance in ROI 6 can be attributed to the higher T_2_ and T_2_* values within the ROI, i.e., the relative variance of the T_2_ and T_2_* values corresponding to ROI 6 does not substantially differ from the relative variances in ROIs 1–5. For ROI 7, the relative variance is increased compared to the other ROIs, which emphasizes the stronger influence of Gibbs ringing and aliasing artifacts in this ROI.

The Bland–Altman plots of the T_2_ values of MS lesions showed that the T_2_ differences follow a negative trend, with increasing mean T_2_ values for higher undersampling factors (R_extra_ = 8–12). This can likely be explained by the reduction of high spatial frequency information in highly undersampled images, leading to smoothing of the lesion sharpness, and reduced contrast between the lesions and surrounding tissue with lower T_2_.

The smaller relative decrease of the adjusted R^2^ of T_2_ and T_2_* with increased undersampling in the phantom study compared to the in vivo studies can be explained by the higher degree of sparsity in the phantom images compared to images of the brain, which promotes accurate recovery of true signal intensities for higher undersampling factors [42]. We observed that the relative decrease of the adjusted R^2^ of T_2_ and T_2_* was lower for the in vivo study with MS patients compared to the healthy volunteers. This can be attributed to the fact that in the case of MS patients, the T_2_ and T_2_* values within all ROIs have a larger range than in the ROIs of the healthy volunteers, and thus the additional variance contributed by the undersampling is proportionally smaller.

A previous study that investigated the performance of R_2_* mapping based on 3D single point imaging (SPI) with CS reconstruction in ex vivo rat tissue and an in vitro cell phantom [15] shows a relative decrease of the R^2^ of about 10–12% for an increase of the undersampling factor from 2 to 10, which is lower than the values observed in our in vivo studies. However, there a number of differences between the two studies that can explain these discrepancies. In the SPI approach, only one k-space point is sampled within a TR, which increases the accuracy of R_2_* measurements, due to a higher sampling rate of the signal decay, but at the expanse of considerably longer scan times. Furthermore, the 3D single point sampling of k-space allows for a higher degree of freedom in the undersampling pattern, which can substantially increase the performance of CS reconstruction at high undersampling factors [36,43]. This motivates the future investigation of 2in1-RARE-EPI using 3D k-space sampling schemes.

The analysis showed that the MAPE of T_2_* is higher and increases more strongly with increasing undersampling compared to the MAPE of T_2_. This can be explained by the hybrid acquisition of the MR signal in 2in1-RARE-EPI, in which the MR signal is first acquired with a RARE module to capture the T_2_ decay, followed by the acquisition with an EPI echo-train to capture the T_2_* decay. This results in a lower SNR of the TE images used for T_2_* mapping, which manifests as an increased MAPE of T_2_*. Notwithstanding this SNR constraint, the overall MAPE of T_2_* was <5% in the phantom studies for R_extra_ ≤ 12, and <8% in the in vivo studies for R_extra_ ≤ 6. The overall MAPE of T_2_ remained <4.4% for R_extra_ ≤ 12 in the phantom and in vivo studies.

Due to the consecutive sampling of the T_2_ and T_2_* decay within each TR, the measurement of T_2_* values could be impaired for tissues exhibiting very low T_2_ values. This could be due, e.g., as a result of increased iron deposition, which is a pathological feature of some neurodegenerative diseases, including MS. The lowest T_2_ values of the tissues investigated in the current study are about 60 ms, which does not impact the T_2_* measurement. Evaluating simultaneous T_2_ and T_2_* mapping with 2in1-RARE-EPI for lower T_2_ values will require further studies, including an adaptation of the length of the RARE and EPI module to achieve optimal results.

In this study, we used a limited slice coverage (*n* = 3, slice thickness = 5 mm) to demonstrate the feasibility of our approach. This could be easily scaled up to meet the needs of clinical applications, by reducing the slice thickness and increasing the total number of slices acquired. Reduced slice thickness would also mitigate partial volume effects and result in a better coverage of lesions. Given the high SNR we observed for the 2in1-RARE-EPI using the imaging parameters of the current study, we expect the results presented here to also hold for lower slice thicknesses. The long TR used in the 2in1-RARE-EPI will allow for acquisition of additional slices without increasing the acquisition time.

The scan time reduction shown in this work will benefit patient compliance, and enhances the robustness to bulk motion. With an extra undersampling factor of R_extra_ = 6 (R_eff_ = 12), the acquisition time for simultaneous T_2_ and T_2_* mapping can be reduced from 07:12 min to 01:39 min. An extra undersampling of R_extra_ = 12 (R_eff_ = 24) permits a scan time as short as 01:06 min. The 2in1-RARE-EPI approach is compatible with the concept of using different undersampling rates for the RARE module and for the EPI module. This approach would permit an improvement of the RF deposition economy of the RARE module. Even further scan time reductions could be achieved by employing simultaneous multi-slice imaging techniques which are compatible with 2in1-RARE-EPI [6,44,45,46].

The current study is a proof-of-concept demonstration of the feasibility of using undersampled 2in1-RARE-EPI in a realistic clinical context, and was not designed or powered to detect group differences. Further clinical studies with larger numbers of patients will be needed to robustly test the sensitivity to group differences.

## 5. Conclusions

We demonstrate that the scan time for simultaneous T_2_ and T_2_* mapping can be substantially reduced, by utilizing high radial undersampling in 2in1-RARE-EPI together with CS-reconstruction. This improvement comes at the cost of only minor reductions of high spatial frequency information, and provides a proof-of-concept technical foundation to support the implementation of quantitative mapping in routine clinical practice. This approach could also be applied to broader clinical studies on the potential use of T_2_ and T_2_* as imaging biomarkers of neuroinflammatory and neurodegenerative diseases, thereby expanding previous work [47,48,49] using these imaging biomarkers. Furthermore, 2in1-RARE-EPI can also be adapted to simultaneously provide temperature maps in addition to T_2_ and T_2_* maps. The potential range of clinical applications for accelerated 2in1-RARE-EPI for T_2_ and T_2_* mapping extends well beyond MS to several other pathologies in the brain and other target organs.

## Data Availability

The data presented in this study are available on reasonable request from the corresponding author.
